# Diffuse FDG uptake in the bilateral lungs: hypersensitivity pneumonitis supported by low-dose CT findings

**DOI:** 10.22038/AOJNMB.2021.56000.1393

**Published:** 2022

**Authors:** Shun Goto, Yohji Matsusaka, Tomohiko Yamane, Yuki Hoshino, Ichiei Kuji

**Affiliations:** 1 Department of Nuclear Medicine, Saitama Medical University International Medical Center, Saitama, Japan; 2 Department of Respiratory Medicine, Saitama Medical University Hospital, Saitama, Japan

**Keywords:** ^18^F-fluorodeoxyglucose, Hypersensitivity pneumonitis, Low-dose computed tomography, Positron emission tomography

## Abstract

Hypersensitivity pneumonitis (HP) is an interstitial lung disease resulting from an immune-mediated response in susceptible and sensitized individuals to various inhaled antigens in the environment. Imaging diagnosis is usually based on high-resolution CT findings. Here, we present a 49-year-old man with a history of diffuse large B-cell lymphoma presented with fever and occasional cough. ^18^F-fluorodeoxyglucose (FDG) positron emission tomography/computed tomography (PET/CT) revealed diffuse FDG uptake in the bilateral lungs. Expiratory low-dose CT simultaneously performed in PET scanning revealed centrilobular nodules and air trapping in ground glass opacities (GGO). Our imaging diagnosis was acute hypersensitivity pneumonitis (HP). Based on the results of his clinical course, blood laboratory tests, and bronchoscopy, he was diagnosed with acute HP. Diffuse pulmonary FDG uptake can be seen in the patients with acute HP. In addition, expiratory low-dose CT findings of centrilobular nodules and air trapping in GGO may be helpful for accurate diagnosis of acute HP.

## Introduction

 Hypersensitivity pneumonitis (HP) is an interstitial lung disease resulting from an immune-mediated response in susceptible and sensitized individuals to a large variety of inhaled antigens found in the environment (1). Diagnosis is usually based on an accurate exposure history, clinical presentation, specific IgG antibodies to the offending antigen, bronchoalveolar lavage (BAL), characteristic high-resolution CT findings, and pathological features. Here, we present the case of a patient diagnosed with acute HP using ^18^F-fluorodeoxyglucose (FDG) positron emission tomography/computed tomography (PET/CT).

## Case report

**Figure 1:**
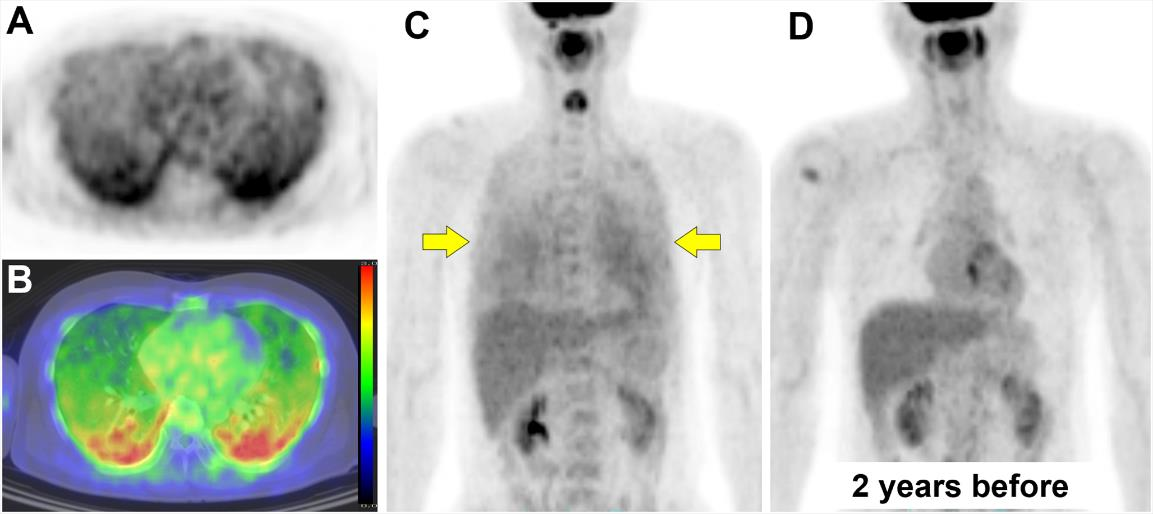
On axial images of PET (**A**) and PET/CT fusion (**B**), dorsal-dominant diffuse pulmonary uptake was confirmed. Maximum intensity projection image showed diffuse uptake in the bilateral lungs (**C**, arrows), which was not confirmed 2 years before (**D**)

 A 49-year-old man, who was treated for diffuse large B-cell lymphoma 3 years before, presented with fever and complained of occasional cough. Test of reverse-transcriptase polymerase chain reaction (RT-PCR) for severe acute respiratory syndrome coronavirus 2 (SARS-COV-2) was negative, and serum soluble interleukin-2 receptor level was elevated (1117 U/mL; upper normal limit, 582 U/mL). He was referred for FDG PET/CT for suspicion of the recurrence of lymphoma. On axial images of PET and PET/CT fusion (Figure 1a and 1b), dorsal-dominant diffuse pulmonary uptake was confirmed. Maximum intensity projection image showed diffuse uptake in the bilateral lungs (Figure 1c, arrows), which was not confirmed 2 years before (Figure 1d). Differential diagnoses of diffuse pulmonary FDG uptake included intravascular lymphoma (2, 3), drug-induced pneumonia (4-6), pneumocystis carinii pneumonia (7), and viral infection (8).

**Figure 2:**
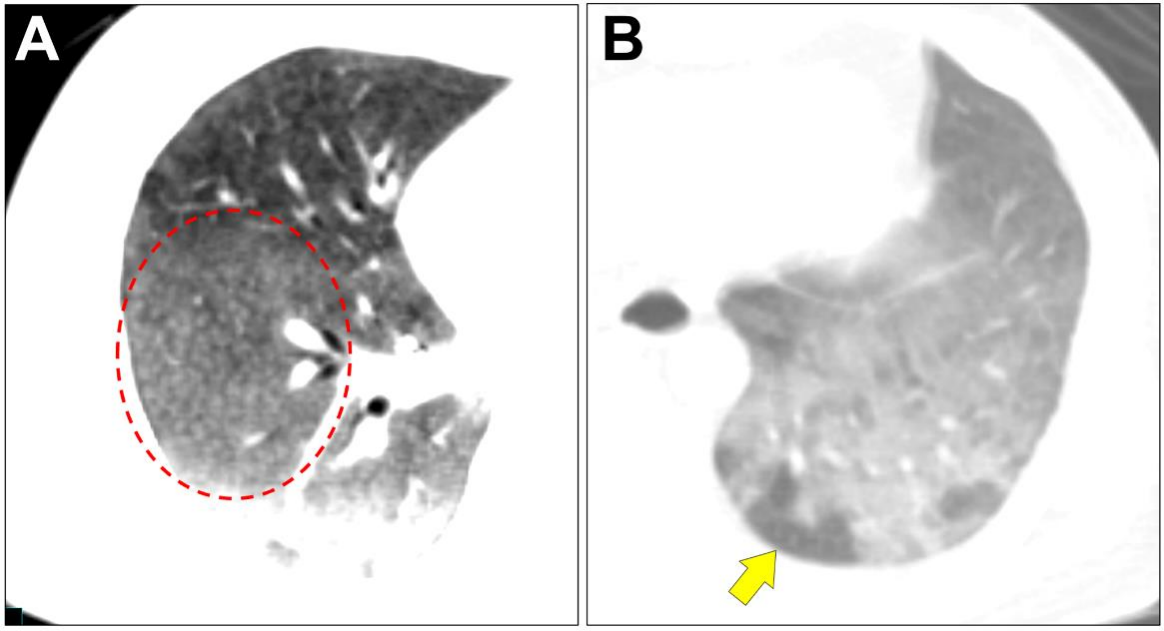
Expiratory low-dose CT simultaneously performed with PET revealed centrilobular nodules (A, red circle) and air trapping (B, arrow) in ground glass opacities

Expiratory low-dose CT simultaneously performed with PET revealed centrilobular nodules (Figure 2a, red circle) and air trapping (Figure 2b, arrow) in ground glass opacities (GGO), which are characteristic of acute hypersensitivity pneumonitis (HP) (9, 10). Our imaging diagnosis was acute HP, and the patient was referred to the pulmonologist. His symptoms started one month before the FDG PET/CT scan and had a history of cleaning moldy part of his own house around the onset period. Serum anti-Trichosporon asahii antibody was positive. The analysis of BAL fluid obtained in bronchoscopy showed remarkably increased lymphocyte ratio (91%; normal range, 10–15%) and normal CD4/CD8 ratio (0.22; normal range, <1.0). Pathological examination of the transbronchial lung biopsy (TBLB) specimen identified inflammatory lesions with non-caseous granulomas and Masson’s bodies. Based on all of these results, a diagnosis of acute HP was confirmed (11). After two weeks of hospitalization, the symptoms gradually subsided by antigen avoidance. Chest CT scan also showed improvement of the GGO.

## Discussion

 We reported the case of acute HP which was detected with FDG PET/CT. Although there are two reports that retrospectively analyzed pulmonary FDG uptake values in a few patients with HP (12, 13), the process of radiological diagnosis is not shown in those reports. To the best of our knowledge, there is only one report about the diagnosis process using radiological findings of HP on FDG PET/CT (14). Compared to the previous case, the present case showed impressive bilateral diffuse pulmonary uptake on the MIP image. Furthermore, centrilobular nodules on low-dose CT were not shown in the previous case and this finding was one of the clues to the precise diagnosis of acute HP.

 Although diagnosis of HP is done based on various clinical data of patients, radiological findings is important. Characteristic CT findings of acute HP includes centrilobular nodules and air trapping in GGO. GGO in the lungs of HP patients is caused by parenchymal infiltration of leucocytes, which is supported by the analysis of BAL fluid and pathological results of TBLB. The pulmonary FDG uptake in HP is likely to reflect metabolic activation of lymphocytes. Therefore, the extend of FDG uptake in the GGO may be useful to differentiate inflammatory GGO from non-inflammatory GGO such as pulmonary edema caused by cardiac failure. However, diffuse pulmonary FDG uptake is not specific for HP and implies several types of differential diagnosis as mentioned above. In the present case, CT findings of air trapping and centrilobular nodules in GGO were clues to the imaging diagnosis. In acute HP, centrilobular nodules and air trapping in GGO are suggestive of inflammation surrounding bronchioles and small airway obstruction, respectively (9). It is notable that these CT findings were clearly confirmed even on low-dose CT images. We used 50 mA of tube current in low-dose CT. In standard-dose CT, 180-200 mA of tube current is generally used, and although the image quality of low-dose CT is inferior to that of standard-dose CT, low-dose CT is acceptable in pulmonary nodules identification (15, 16). In the present case, although the image quality of low-dose CT was not high, we identified the key findings of HP. Furthermore, air trapping was recognizable on the expiratory low-dose CT. Since CT scanning in PET/CT imaging is often performed under forced expiratory phase, CT images are suitable for evaluating air trapping. Therefore, acute HP was diagnosable by evaluating the expiratory low-dose CT in PET/CT.

 The clinical value of pulmonary FDG uptake in the patients with HP is still unknow. The pulmonary uptake in interstitial pneumonia or radiation-induced pneumonia is reported to be useful to quantify the extend of pulmonary inflammation (17, 18). Pulmonary FDG uptake in HP may be correlated with the extend of pulmonary inflammation. Because chronic HP is important complication of acute HP (1), pulmonary FDG uptake in the patients with acute HP might have prognostic value or predictor of progression from acute to chronic HP (14). Further investigation is needed to assess the utility of FDG PET/CT in patients with HP.

## Conclusion

 We presented the case of acute HP with diffuse pulmonary FDG uptake. The diagnosis was achieved by carefully evaluating the expiratory low-dose CT findings. Nuclear medicine physicians should know that, when diffuse pulmonary uptake of FDG is noted, a detailed interpretation of low-dose CT findings in PET/CT may help in the differential diagnosis of acute HP.

## Conflict of interest

 None of the author reports any conflict of interest.

## Grant support for the research reported

 No grant support was received for this report.
